# Data on measurement of the strain partitioning in a multiphase Zn-Al eutectic alloy

**DOI:** 10.1016/j.dib.2018.09.010

**Published:** 2018-09-08

**Authors:** Zhicheng Wu, Stefanie Sandlöbes, Jing Rao, James S.K.L. Gibson, Benjamin Berkels, Sandra Korte-Kerzel

**Affiliations:** a Institute of Physical Metallurgy and Metal Physics, RWTH Aachen University, D-52056 Aachen, Germany; b Aachen Institute for Advanced Study in Computational Engineering Science (AICES), RWTH Aachen University, D-52056 Aachen, Germany

## Abstract

This paper presents original data related to the research article “Local mechanical properties and plasticity mechanisms in a Zn-Al eutectic alloy” (Wu et al., 2018). The raw data provided here was used for in-situ digital image correlation on the microstructural level using a new method described in the related study. The data includes sample preparation details, image acquisition and data processing. The described approach provides an approach to quantify the local strain distribution and strain partitioning in multiphase microstructures.

**Specifications table**

**Table:** 

Subject area	*Physics*
More specific subject area	Materials science
Type of data	*Micrographs and tables*
How data was acquired	*FEI Helios Nanolab 600i scanning electron microscope at an acceleration voltage of 3* *kV with an in-lens secondary electron (SE) detector*
Data format	*Raw data*
Experimental factors	*A monolayer of SiO*_*2*_*particles with average particle size of 40 nm was dispersed on the specimen surface.*
Experimental features	*Quasi in-situ tensile tests were performed at 85* °*C at a constant strain rate of* 5 × 10^−^^4^ s^−1^*. The tests were interrupted at 2% and 5% global engineering strain.*
Data source location	*Institute of Physical Metallurgy and Metal Physics, RWTH Aachen University, D-52056 Aachen, Germany*
Data accessibility	*With this article and on Mendeley:*10.17632/y269sfgw6m.1
Related research article	[1] Z. Wu, S. Sandlöbes, R. Jing, J.S.K.L. Gibson, B. Berkels, S. Korte-Kerzel, *Local mechanical properties and plasticity mechanisms in a Zn-Al eutectic alloy*, Materials and Design, 157 (2018) 337–350.

**Value of the data**

• The data shows quantitative and qualitative descriptions of the local strain partitioning in a multiphase Zn-Al eutectic alloy, which may give indications for the strain partitioning in other multicomponent materials.
• Combination of DIC and SEM enables the measurement of strain partitioning at a very high resolution.
• The data provides a water-free method of how to deposit SiO_2_ particles suitable for DIC measurements on a specimen surface.
• The particle disposition method may be expanded to other materials that are reactive with water.

## 1 Data

**Fig. 1:**
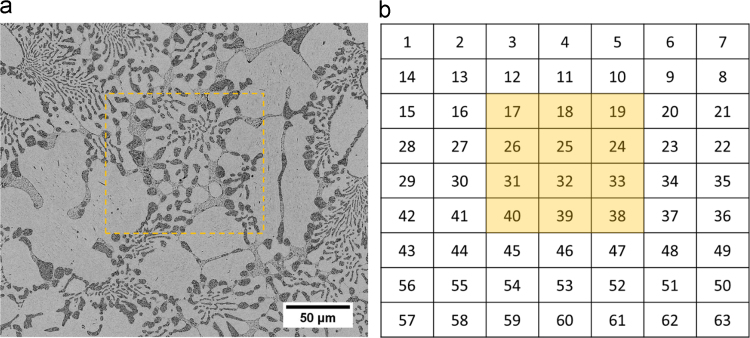
(a) Backscattered electron (BSE) micrograph of the whole region of interest (ROI) in alloy ZnAl4Cu1Mg0.31; (b) schematic presentation of the position of individual images in the ROI. The orange boxes indicate the areas shown in Fig. 2.

**Fig. 2:**
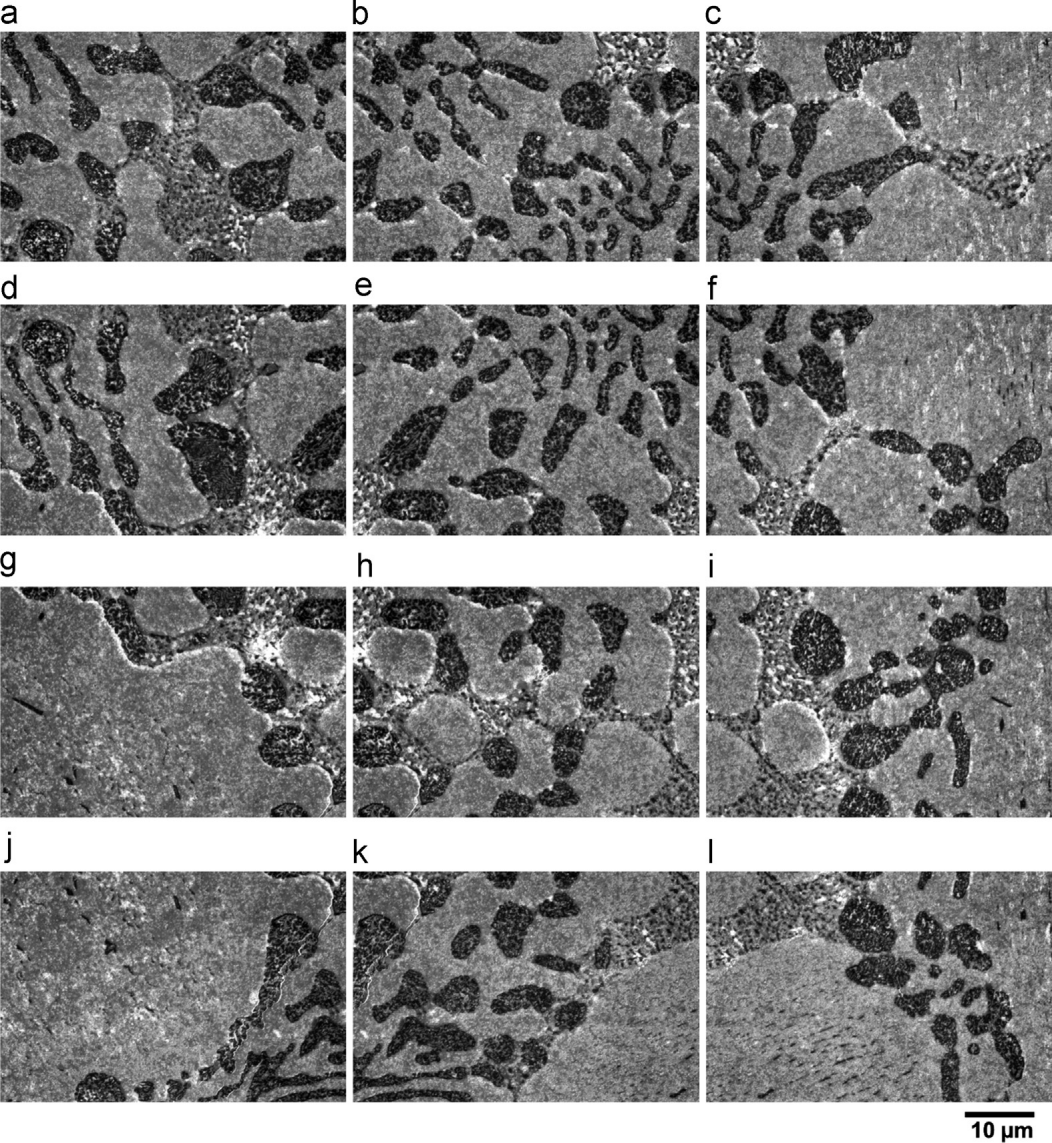
High magnification in-lens SE images in the region of interest (ROI) in alloy ZnAl4Cu1Mg0.31 after 2% global strain at positions (a–c) 17–19; (d–f) 26–24; (g–i) 31–33 and (j–l) 40–38. The locations are illustrated in Fig. 1. The scale bar of all images is given below (l).

This data set contains 189 high-resolution in-lens SE micrographs of a ZnAl4Cu1Mg0.31 (wt%) specimen showing the microstructural evolution during tensile deformation. At each deformation step, the same region of interest (ROI) was observed using SEM. Fig. 1a shows a backscattered electron (BSE) overview micrograph of the region of interest (ROI) prior to deformation. To ensure sufficiently high resolution of the micrographs, the ROI was divided into subsets of 7 × 9 micrographs, as described in Table 1. Fig. 1b shows the spatial distribution of these individual micrographs. Fig. 2 presents individual SE micrographs in the region of interest.

**Table 1:** File structure of dataset.

Data element	Description
Directory: 0percent	SE images acquired prior to deformation at 0% global strain
File name	
01_0.tif	SE micrograph of position 1 prior to deformation at 0% global strain
02_0.tif	SE micrograph of position 2 prior to deformation at 0% global strain
…	…
63_0.tif	SE micrograph of position 63 prior to deformation at 0% global strain
Directory: 2percent	SE images acquired at 2% global strain
File name	
01_1.tif	SE micrograph of position 1 at 2% global strain
02_1.tif	SE micrograph of position 2 at 2% global strain
…	…
63_1.tif	SE micrograph of position 63 at 2% global strain
Directory: 5percent	SE images acquired at 5% global strain
File name	
01_2.tif	SE micrograph of position 1 at 5% global strain
02_2.tif	SE micrograph of position 2 at 5% global strain
…	…
63_2.tif	SE micrograph of position 63 at 5% global strain

## 2 Experimental design, materials, and methods

### 2.1 SiO_2_ particles speckle deposition

**Fig. 3:**
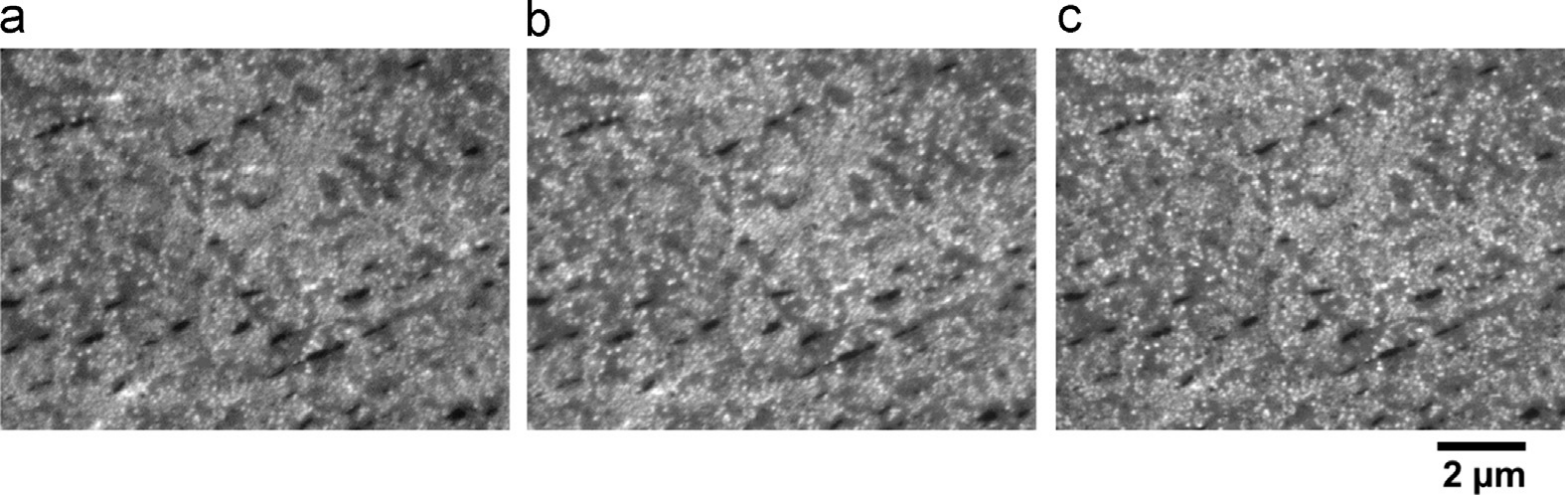
Enlarged in-lens SE images showing SiO_2_ particle pattern on the sample surface at (a) 0%, (b) 2% and (c) 5% global strain. The scale bar of all images is given below (c).

µ-DIC measurements were performed using dog-bone shaped specimens with a gauge length of 3.56 mm and a centre cross-section of 1 × 1.5 mm^2^. A monolayer of SiO_2_ particles with an average particle size of 40 nm was dispersed on the specimen surface, Fig. 3. Table 2 gives the detailed procedure of pattern deposition, all steps were performed water-free.

**Table 2:** Detailed procedures for depositing the SiO_2_ nanoparticles on the sample surface.

Step	Procedure	Purpose
1	Preparation of the specimen using standard metallographic procedures up to 50 nm gamma alumina finish.	Obtaining a flat and deformation-free surface.
2	Preparation of an OPS suspension (SiO_2_ particles, Struers®) in ethanol with a ratio of 1:3 (SiO_2_:Ethanol) using an ultrasonic bath.	Homogeneous distribution of SiO_2_ particles in the suspension.
3	Dipping of the specimen in the suspension for 30 s in ultrasonic bath.	Deposition of SiO_2_ particles on specimen surface with a homogeneous distribution to avoid accumulation of particles.
4	Rinsing the sample with ethanol spray, and subsequent drying of the sample with an air fan.	Removal of floating particles, formation of a monolayer of SiO_2_ particles on the sample surface.

### 2.2 Image acquisition

A universal electromechanical testing machine (DZM) was used to deform a sample with deposited SiO_2_ particles at 85 °C and a constant strain rate of 5 × 10^−4^ s^−1^ to 2% and 5% global engineering strain. SE micrographs of the samples were obtained prior to deformation and after 2% and 5% global strain, respectively, at an FEI Helios Nanolab 600i scanning electron microscope. To achieve micrographs suitable for µ-DIC processing, the images were achieved with an in-lens SE detector, a low beam voltage of 3 kV and a small working distance of 3 mm [2], [3]. A region of interest with an area of 300 × 255 µm^2^ was identified prior to deformation and divided into 7 × 9 subsets each having a resolution of 1536 × 1024 pixels (51.8 × 34.5 µm) with 20% overlapping between two adjacent images in both horizontal and vertical directions, Fig. 1.

### 2.3 µ-DIC processing

We applied two different approaches of calculating the local strain distribution and strain partitioning from the deformation microstructures based on (i) the commercial software GOM Correlate, and (ii) non-rigid image registration.

The images were analysed using the software GOM Correlate (V8.1, GOM mbH) with a facet size of 17 × 17 pixels and a point distance of 10 pixels, which corresponds to a facet size of around 0.5 × 0.5 µm^2^, and an overlap of 40% between the facets.

Non-rigid image registration was applied as an alternative approach to estimate the local deformation. To register a deformed image to the original undeformed image, we used the multi-level non-rigid registration approach as described in [4]. Instead of the Dirichlet energy used in [4], we used a hyperelastic regulariser [5] to account for the highly non-linear structure of the local material deformation. This also required the replacement of the Gauss quadrature by a Simpsons quadrature to ensure that the energy stays finite when the estimated solution is prolongated from one level to the next, finer level. In addition, the approach described in [4] was generalised to non-dyadic grids (a dyadic grid is a quadratic grid with 2*^k^* nodes in each coordinate direction) following [6], i.e. the full resolution data were downsampled to the largest dyadic grid that is smaller than the full data grid. Then, the registration algorithm for the dyadic case was applied. Its result was resampled to the full data grid and used there as the initial value. Finally, on the full resolution grid, the minimisation was done using the Quasi Newton BFGS algorithm instead of a regularized gradient descent.

The 2-D equivalent von Mises strain was calculated using Eq. (1) [7]:

(1) $$\varepsilon_{\mathrm{eq}}=\sqrt{\varepsilon_{\mathrm{xx}}^{2}+{2\varepsilon }_{\mathrm{xy}}^{2}{+\varepsilon }_{\mathrm{yy}}^{2}}$$

Assuming (u,v) are the components of displacement of an arbitrary point in *x*- and *y*-directions, the strains $\varepsilon_{\mathrm{xx}}$ , $\varepsilon_{\mathrm{yy}}$ and $\varepsilon_{\mathrm{xy}}$ were obtained through Eqs. (2), (3), (4) [8]:

(2) $$\varepsilon_{\mathrm{xx}}=\frac{\partial u}{\partial x}+\frac{1}{2}[({\frac{\partial u}{\partial x})}^{2}+({\frac{\partial v}{\partial x})}^{2}]$$

(3) $$\varepsilon_{\mathrm{yy}}=\frac{\partial v}{\partial y}+\frac{1}{2}[({\frac{\partial u}{\partial y})}^{2}+({\frac{\partial v}{\partial y})}^{2}]$$

(4) $$\varepsilon_{\mathrm{xy}}=\frac{1}{2}\left(\frac{\partial u}{\partial y}+\frac{\partial v}{\partial x}\right)+\frac{1}{2}(\frac{\partial u}{\partial x}\frac{\partial u}{\partial y}+\frac{\partial v}{\partial x}\frac{\partial v}{\partial y})$$
